# Prognostic impact of residual SYNTAX score in patients with obstructive sleep apnea and acute coronary syndrome: a prospective cohort study

**DOI:** 10.1186/s12931-019-1008-z

**Published:** 2019-02-28

**Authors:** Yaping Zeng, Shuhan Yang, Xiao Wang, Jingyao Fan, Shaoping Nie, Yongxiang Wei

**Affiliations:** 1 Emergency & Critical Care Center, Beijing Anzhen Hospital, Capital Medical University, 2nd Anzhen Road, Chaoyang District, Beijing, 100029 China; 2 Department of Cardiology, Xinxiang Central Hospital, Xinxiang, Henan Province People’s Republic of China; 3 Department of Otolaryngology Head & Neck Surgery, Beijing Anzhen Hospital, Capital Medical University, 2nd Anzhen Road, Chaoyang District, Beijing, 100029 China

**Keywords:** Obstructive sleep apnea, Residual SYNTAX score, Acute coronary syndrome

## Abstract

**Background:**

There is a paucity of data from large prospective study evaluating the prognostic significance of the residual Synergy between percutaneous intervention with Taxus drug-eluting stents and cardiac surgery (SYNTAX) Score (rSS) in patients with obstructive sleep apnea (OSA) and Acute Coronary Syndrome (ACS).

**Methods:**

ACS patients who undergoing percutaneous coronary angiography and completing a sleep study during hospitalization were prospectively enrolled. The baseline SYNTAX Score (bSS) and the rSS after revascularization were assessed. Complete revascularization (CR, rSS = 0) and incomplete revascularization (ICR, rSS > 0) were categorized. OSA (apnea hypopnea index, AHI ≥ 15) and non-OSA (AHI < 15) were grouped according to AHI. The primary endpoint of the study was major adverse cardiovascular and cerebrovascular events (MACCEs), defined as a composite of cardiovascular death, myocardial infarction, stroke, ischemia-driven revascularization, or hospitalization for UAP or heart failure.

**Results:**

Overall, 752 patients were prospectively enrolled. At a median follow-up of 1 year, the incidence of MACCEs was significantly higher in the OSA than in the non-OSA group (hazard ratio [HR]:1.68; 95% confidence interval [CI]:1.04–2.72; *P* = .034). ICR was associated with a higher risk of MACCEs in the non-OSA group (HR:3.34;95% CI:1.0–11.12; *P* = .05). The OSA patients with ICR had a 5.1 higher risk of MACCEs compared with the non-OSA with CR group, *P* = .007. The OSA patients with CR had a similar 1-year MACCEs as all the non-OSA patients (HR:1.10; 95% CI:0.515–2.349; *P* = 0.806).

**Conclusions:**

ACS patients with OSA and ICR have a high rate of MACCEs at 1 year. In contrast, the prognosis of ACS patients with OSA but CR is favorable and similar to patients without OSA. Adequate level of revascularization is recommended to optimize clinical outcomes in ACS patients with OSA.

**Trial registration:**

Clinicaltrials.gov identifier NCT03362385.

## Backgrounds

OSA (Obstructive Sleep Apnea) characterized by repeated hypoxemia at night, may promote the occurrence and development of cardiovascular disease through the activation of sympathetic system, inflammatory reaction, oxidative stress and metabolic abnormalities [1–3]. OSA has been advocated as a new emerging independent risk factor for coronary artery disease by America expert consensus documents and Europe clinical guidelines [4–6]. It has been confirmed recently in a large prospective cohort study [7]. Intravascular Ultrasound has further demonstrated those patients with OSA present greater plaque burden [8]. However, the relation between cardiovascular outcomes and OSA still remain debatable even in randomized trials [4, 9, 10].

The Synergy between percutaneous intervention with Taxus drug-eluting stents and cardiac surgery (SYNTAX) score has been developed to stratify the complexity and severity of coronary lesions [11]. Residual SYNTAX Score (rSS) was derived from SYNTAX score to quantify the burden of residual coronary artery disease after percutaneous coronary intervention (PCI) or coronary artery bypass graft (CABG), and has been validated as an independent predictor for clinical adverse events [12–14]. However, studies examining the impact of rSS on patients with OSA and acute coronary syndrome (ACS) have not yet been elucidated.

The objective of the present study was to evaluate the prognostic impact with rSS in patients with OSA and ACS in a prospective single center cohort study.

## Methods and materials

### Study design and subjects

The OSA-ACS project (NCT03362385) was a prospective, single-center, observational cohort study that recruited consecutive patients with ACS diagnosis at Beijing An Zhen Hospital, Capital Medical University, Beijing, China from May 2015 to June 2017. A total of 899 eligible ACS patients who have undergone overnight portable sleep study were eligible for inclusion. ACS includes ST-segment elevation myocardial infarction (STEMI), none ST-segment elevation myocardial infarction (NSTEMI), and unstable angina pectoris (UAP). Inclusion criteria: i) age ≥ 18 years old and < 85 years old. ii) ACS. Exclusion criteria: i) cardiogenic shock; ii) cardiac arrest; iii) history of malignancy; iv) failed sleep study; v) failure of coronary angiography or poor quality; vi) central sleep apnea; vii) known OSA and continuous positive air pressure (CPAP) treatment. The study was approved by the Institutional Review Board of Beijing Anzhen Hospital, Capital Medical University and all patients provided written informed consent.

### Overnight level 3 polygraphy and definitions

The sleep studies were conducted with a portable diagnostic device (Apnealink Air, Resmed, Australia) for more than 7 h during the night after admission by a trained research team. Nasal airflow, thoracic-abdominal movements, arterial oxygen saturation, heart rate, and snoring episodes were recorded. According to apnea hypopnea index (AHI), patients were categorized to 2 groups: OSA group (AHI ≥ 15 events/h), non-OSA group (AHI < 15 events/h). For the patients with moderate to severe sleep apnea (AHI ≥ 15), particularly those with excessive daytime sleepiness, we referred them to sleep center for further evaluation and consideration of CPAP therapy.

### Procedure

The decision of PCI strategy and stenting were left to the discretion of the operator. Dual antiplatelet therapy with aspirin and P2Y_12_ receptor inhibitor (i.e. clopidogrel or ticagrelor) were recommended for at least 12 months according to the current standard guidelines. All patients were followed up routinely in outpatients or by phone at 1 month, 3 months, 6 months, 12 months, and every 6 months thereafter.

### SYNTAX SCORE assessment

Basic SYNTAX Score (bSS) and rSS were calculated by 2 experienced interventional cardiologists who were blinded to the clinical outcomes and the sleep patterns of the patients using a web-based calculator (www.syntaxscore.com), and disagreement was resolved by consensus. Lesions with > 50% diameter stenosis in vessel ≥1.5 mm in diameter were scored using the SS algorithm described previously.

The rSS was defined as the remaining SYNTAX Score after PCI or CABG. In the case of staged PCI procedures, the final planned procedure was used to calculate the point for rSS. For the patients who underwent CABG, the rSS was scored by comparing the bSS with the surgical procedure reports, the same, the final decision was made by consensus in cases of disagreement between the 2 cardiologists. Moreover, each vessel disease was still scored in terms of vessel characteristics (tortuosity, severe calcification) or any residual lesions distal to the graft allocation [14]. Patients were then classified into 2 groups based on the points of rSS: complete revascularization (CR): rSS = 0; incomplete revascularization (ICR): rSS > 0 [15].

The intra-observer variability of the bSS and rSS using the intraclass correlation analysis were 1.0 (0.99–1.0), *P* < .001 and 0.98 (0.95–0.99), *P* < .001. The inter-observer variability of the bSS and rSS was 1.0 (0.99–1.0) and 0.99 (0.99–0.99) respectively, both *P* < .001.

### The primary endpoints and definitions

The primary endpoint of the study was major adverse cardiovascular and cerebrovascular events (MACCEs), defined as a composite of cardiovascular death, myocardial infarction, stroke, ischemia-driven revascularization, or hospitalization for UAP or heart failure.

### Statistical analysis

Continuous data were expressed as mean ± standard deviation (SD) and compared using the Student t test. The chi-square or Fisher’s exact test was used for comparison of categorical variables. Clinical outcomes were determined using Kaplan-Meier method and compared using the log-rank test. Cox multivariate regression analysis was used to determine the independent predictor of clinical events, with variable entry/stay criteria of 0.1/0.1. Interclass correlation coefficient (ICC) was used to assess the inter-observer (Zeng Y and Yang S) and intra-observer agreement of bSS, as well as rSS after PCI or CABG with 95% confidence intervals. An ICC < 0.4 indicates bad agreement, an ICC between 0.4 and 0.75 indicates moderate agreement, and ICC values > 0.75 indicates excellent agreement [16]. A *P* value < .05 were considered statistically significant. Statistical analyses were performed using SPSS version 22 (IBM Corp, Armonk, NY).

## Results

### Study population and case selection

**Fig. 1:**
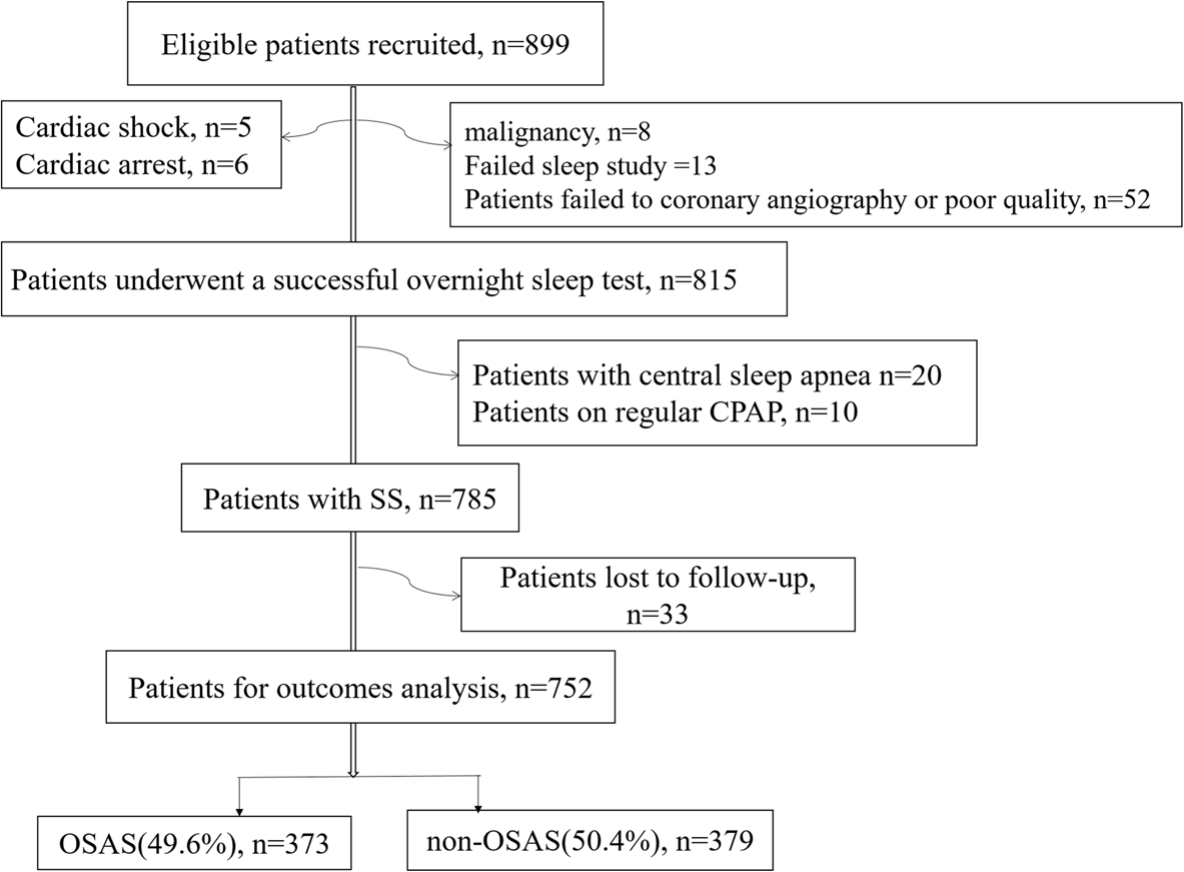
Study flow chart

The study flowchart was shown in Fig. 1. From May 2015 to June 2017, 752 eligible patients were prospectively included in the analysis. Overall, mean age was 57.2 ± 10.2 years, 82.6% were male and 30.6% were diabetes. STEMI, NSTEMI, UAP according to the Braunwald classification were the clinical presentation in 32.3, 25.1 and 42.6% of the population respectively. The mean bSS and rSS was 14.6 ± 11.4 and 7.6 ± 8.7.

The cohort’s baseline demographic and clinical characteristics were listed in Table 1. OSA was observed in 49.6% of the population. Mean oxygen saturation (SO_2_) in the OSA (93.0 ± 2.3%) was lower than in the non-OSA group (94.6 ± 1.7%, *P* < .001). Interestingly, multi-vessel diseases were more frequently observed in the OSA than in the non-OSA cases (three vessel disease: 27.3% vs 22.7%; two vessel disease: 38.3% vs 32.2%, overall *P* = .024). Similarly, chronic total occlusion lesions (CTO) in the OSA (23.6%) was more than in the non-OSA group (15.3%), *P* = .004. Finally, more stents (1.0 ± 1.0) were implanted in the OSA than in the non-OSA group (0.8 ± 1.0), *P* = .046.

**Table 1:** Baseline patient characteristics

	OSA (*N* = 373)	non-OSA (*N* = 379)	*P* value
Age, years (mean ± SD)	57.6 ± 10.4	56.8 ± 10.0	0.299
Male, n (%)	315, 84.5%	306, 80.7%	0.211
BMI	27.6 ± 3.4	25.8 ± 3.3	< 0.001
AHI	33.7 ± 15.1	7.1 ± 4.0	< 0.001
bSS	14.9 ± 11.0	14.4 ± 11.8	0.350
rSS	7.8 ± 8.3	7.4 ± 9.1	0.054
Hypertension requiring medication, n (%)	255, 68.4%	239, 63.1%	0.144
Hyper cholesterolemia requiring medication, n (%)	103, 27.6%	98, 25.9%	0.621
Diabetes mellitus requiring medication, n (%)	112, 30%	118, 31.1%	0.752
Myocardial infarction history, n (%)	57, 15.3%	52, 13.7%	0.605
Cardiac Intervention history, n (%)	78, 20.9%	54, 14.2%	0.017
Time to last cardiac intervention, (days)	57 ± 49.6	67 ± 75	0.837
Current smokers, n (%)	237, 63.5%	247, 65.2%	0.649
Family history of CAD, n (%)	31, 8.3%	26, 6.9%	0.492
Minimal SO_2,_ %	86.4 ± 4.5	79.4 ± 8.6	< 0.001
Mean SO_2,_ %	93.0 ± 2.3	94.6 ± 1.7	< 0.001
ODI	33.1 ± 16.1	7.9 ± 5.2	< 0.001
Clinical presentation			0.523
STEMI, n (%)	124, 33.2%	119, 31.4%	
NSTEMI, n (%)	87, 23.3%	102, 26.9%	
Unstable Angina Pectoris, n (%)	162, 43.4%	158, 41.7%	
LVEF,%	59.5 ± 7.7	59.8 ± 8.0	0.605
Post-procedural medication, n (%)			
ACE inhibitors or ARB	271, 72.7%	258, 68.1%	0.175
Beta-blockers	290, 77.7%	284, 74.9%	0.391
Antiplatelet therapy			
Aspirin	354, 94.9%	354, 93.4%	0.438
P2Y12 inhibitor	342, 91.7%	331, 87.3%	0.057
Statin	357, 95.7%	356, 93.9%	0.324
Anatomical characteristics			
Multi-vessel disease, n (%)			0.024
Three vessel disease, n (%)	102, 27.3%	86, 22.7%	
Two vessel disease, n (%)	143, 38.3%	122, 32.2%	
Left main disease, n (%)	12, 3.2%	17, 4.5%	0.45
Any total occlusions	88, 23.6%	58, 15.3%	0.004
Any bifurcation	210, 61.4%	198, 58.8%	0.764
Any aorta-ostial lesion			
Diffuse disease			
Any lesion > 20 mm	90, 24.1%	78, 20.6%	0.256
Any severe calcification	29, 7.8%	41, 10.8%	0.168
Any severe tortuosity	32, 8.6%	22, 5.8%	0.159
Any angiographically visible thrombus	53, 14.2%	63, 16.6%	0.366
Procedure related characteristics			
CABG, n (%)	34, 9.1%	41, 10.8%	0.467
PCI, n (%)	58.7%	51.7%	0.057
Total number of stent, n	1.0 ± 1.0	0.8 ± 1.0	0.046
Hospital stay, n	4.9 ± 2.9	4.7 ± 2.9	0.371

Values are shown in mean ± SD, or n (%)

*OSA* obstructive sleep apnea; BMI = body mass index, *AHI* apnea hypopnea index, *bSS* baseline syntax score, *rSS* residual syntax score, *CAD* coronary artery disease, *Mini SO2* minimal oxygen saturation, *Mean SO2* mean oxygen saturation, *ODI* oxygen desaturation index, *STEMI* ST-segment elevation myocardial infarction, *NSTEMI* none ST-segment elevation myocardial infarction, *LVEF* left ventricular ejection fraction, *ACE* angiotensin converting enzyme, *ARB* angiotensin receptor blocker, *CABG* coronary artery bypass graft, *PCI* percutaneous coronary intervention

### The correlation and distribution of the bSS and the rSS

**Fig. 2:**
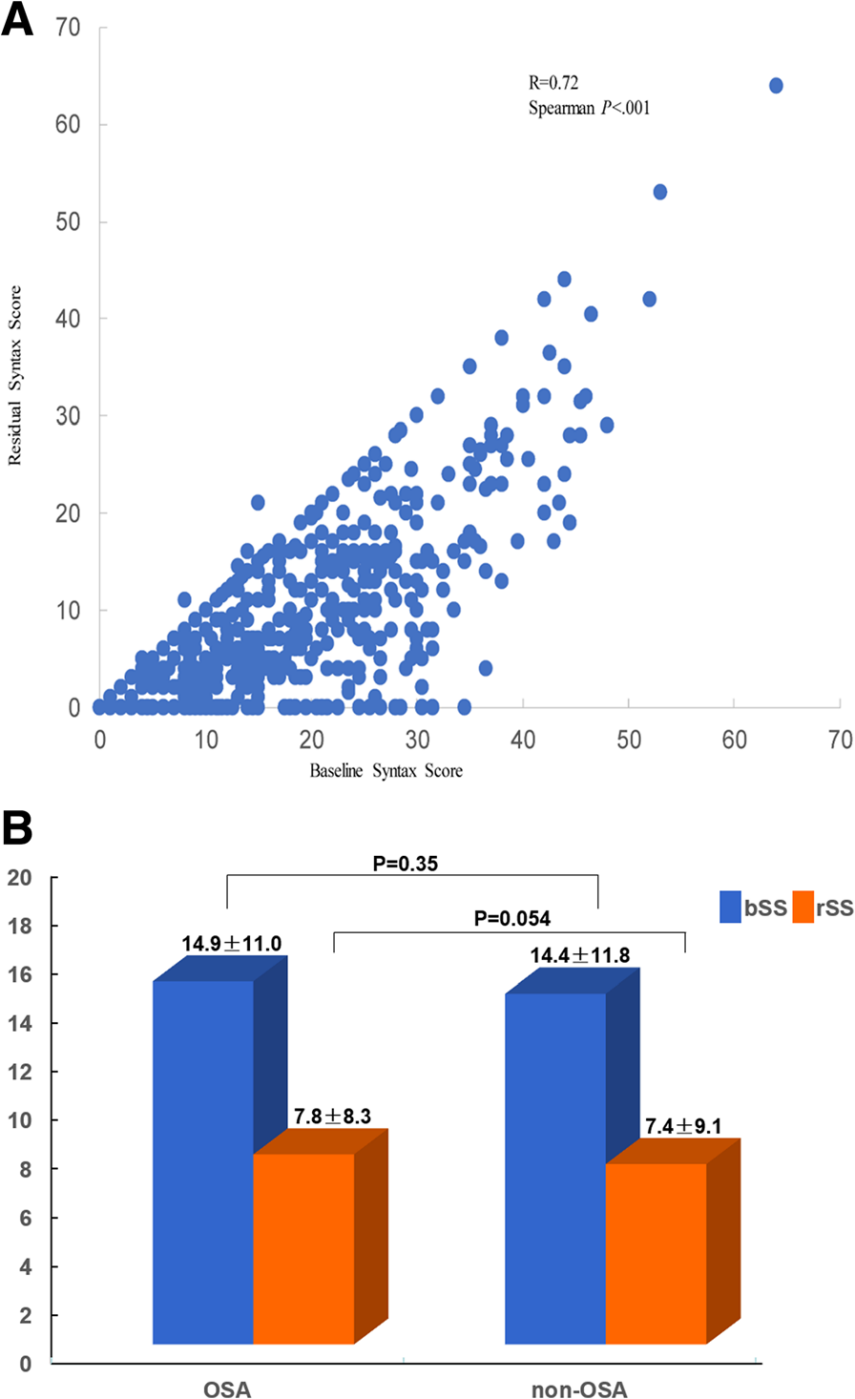
**a**: Correlation between the baseline and the residual SYNTAX Score. Relationship between the baseline (x-axis) and residual (y-axis) SYNTAX Score after angiography in 752 patients. A strong positive correlation was present between the baseline and the residual SYNTAX Score. **b**: Column bars of the baseline and residual SYNTAX Score in the OSA and non-OSA groups. SS = SYNTAX Score; OSA s = Obstructive Sleep Apnea; non-OSA = non-Obstructive Sleep Apnea

The correlation and distribution of the bSS and the rSS were showed in Fig. 2a. The bSS was strongly positively correlated with the rSS (R = 0.72, *p* < 0.001). The frequency of patients with ICR increased across the bSS, while the frequency of CR decreased across the bSS.

### Clinical outcomes

The mean follow-up was 1 year. The total MACCEs rate was significantly higher in the OSA than in the non-OSA patients (12.3% vs 6.9%; hazard ratio [HR]: 1.68; 95% confidence interval [CI]: 1.04–2.72; *P* = .034). However, there were no difference in the rate of ischemia-driven revascularization between the OSA and non-OSA groups (16, 4.3% vs 10, 2.6%, *p* = 0.215).

**Fig. 3:**
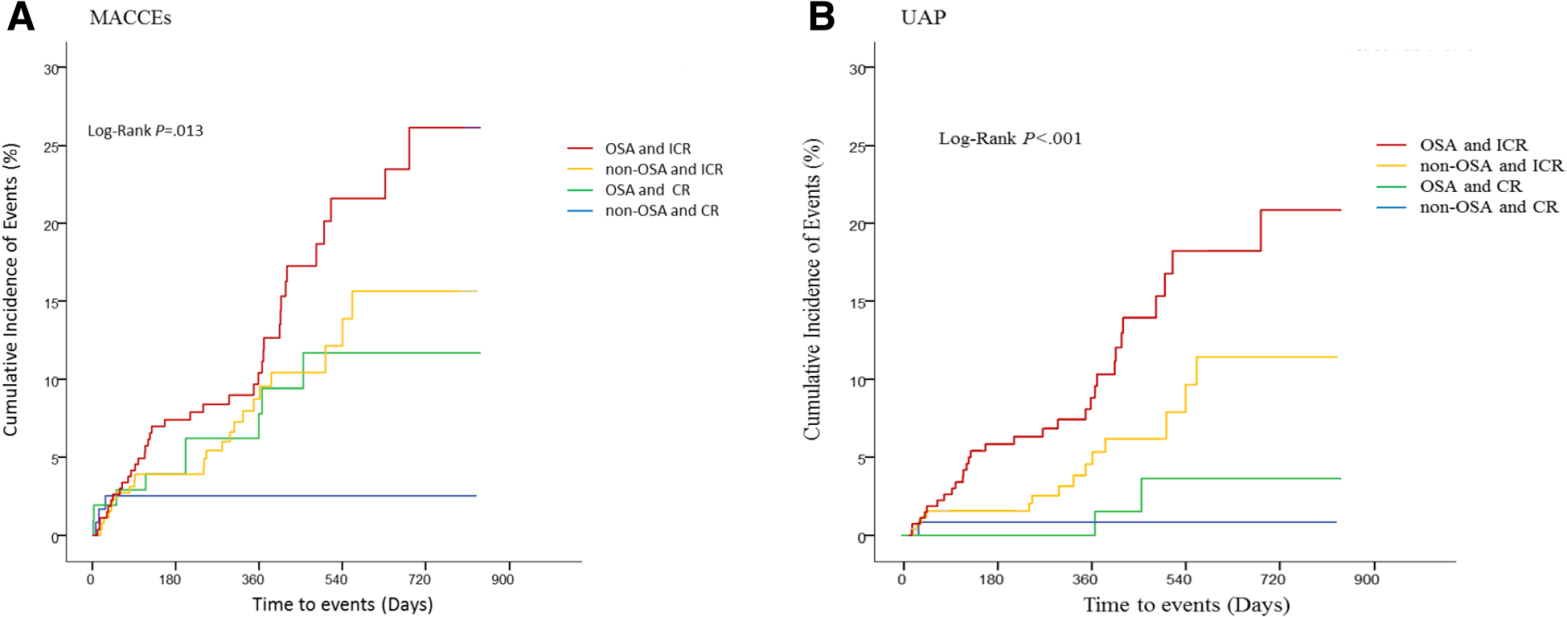
Kaplan-Meier curve showing cumulative events at follow-up in subgroup analysis: (**a**) MACCEs in the OSA patients and ICR, non-OSA and ICR, OSA and CR, non-OSA patients and CR. **b** Unstable angina pectoris in the OSA patients and ICR, non-OSA and ICR, OSA and CR, non-OSA and CR. MACCEs = major adverse cardiovascular and cerebrovascular events; UAP = unstable angina pectoris; OSA = Obstructive Sleep Apnea; non-OSA = non-Obstructive Sleep Apnea; CR = complete revascularization; ICR = incomplete revascularization

Table 2 showed the different clinical outcomes at follow-up for subgroups of the rSS. ICR was associated with a higher risk of MACCEs in the non-OSA group (HR: 3.34; 95% CI: 1.0–11.12; *P* = .05). ICR had a 5.1 higher risk of MACCEs at follow-up compared with the non-OSA with CR group, *P* = .007. Figure 3 demonstrated that the OSA patients with CR (9/104,8.7%) had a higher 1-year MACCEs compared to the non-OSA and CR patients (3/102, 2.5%), (HR: 3.0; 95% CI: 0.79–11.42; *P* = 0.11), the *p* value was not significant. Interestingly, the OSA patients with CR had a similar 1-year MACCEs as all the non-OSA patients (HR: 1.10; 95% CI: 0.515–2.349; *P* = 0.806). There was also no significant difference in the 1-year MACCEs rate in the OSA and in the non-OSA patients with CR (HR: 3.1; 95% CI: 0.84–11.5; *P* = 0.09). The MACCEs in all patients was mainly driven by hospitalization for UAP. In patients with OSA, the rate of hospitalization for UAP was higher in the ICR than in the CR subgroup (HR: 6.73; 95% CI: 1.61–28.2; *P* = .009). The same was also true in the non-OSA group (HR: 6.0; 95% CI: 0.79–45.6; *P* = .084), although the *P* value was not significant.

**Table 2:** Incidence of MACCEs stratified for residual Syntax Score groups

	OSA (N = 373)				Non-OSA (N = 379)			
	CR (*N* = 104)	ICR (*N* = 269)	HR (95% CI)	P value	CR (*N* = 120)	ICR (*N* = 259)	HR (95% CI)	*P* value
Cumulative MACCEs, n (%)	9, 8.7%	37, 13.8%	1.77 (0.85, 3.67)	0.126	3, 2.5%	23, 8.9%	3.34 (1.0, 11.12)	0.05
Cardiovascular death, n (%)	2, 1.9%	1, 0.4%	_	0.177	0	5, 1.9%	_	0.385
Myocardial infarction, n (%)	0	4, 1.5%	_	0.447	2, 1.7%	4, 1.5%	_	0.866
Stroke, n (%)	3, 2.9%	2, 0.7%	_	0.154	0	2, 0.8%	_	0.571
Ischemia- driven revascularization	0	16, 5.9%	_	0.119	0	10, 3.9%	_	0.216
UAP	2, 1.9%	30, 11.2%	6.73 (1.61, 28.2)	0.009	1, 0.8%	14, 5.4%	6.0 (0.79, 45.6)	0.084
Heart failure	2, 1.9%	0	_	0.451	1, 0.8%	2, 0.8%	_	0.891

Values are presented as n, %. Rates are Kaplan-Meier estimates

*OSA* Obstructive Sleep Apnea, *CR* complete revascularization, *ICR* Incomplete revascularization, *HR* Hazard ratio, *CI* confidence interval, *MACCEs* major adverse cardiovascular and cerebrovascular events, *UAP* unstable angina pectoris

### Multivariate cox regression analysis

Multivariate analysis demonstrated that OSA (HR: 1.65; 95% CI: 1.02–2.67, *P* = .044) and rSS (HR: 2.04; 95% CI: 1.09–3.8, *P* = .025) were two independent predictors of the MACCEs at follow-up (Table 3).

**Table 3:** Predictors of MACCE with Univariate and Multivariate Cox Regression Analyses, residual SYNTAX Score was drawn mandatory into the model

	Univariate regression analysis		Multivariate regression analysis	
	Hazard Ratio (95% CI)	*p*-value	Hazard Ratio HR (95% CI)	*p*-value
OSA	1.68 (1.04, 2.72)	0.034	1.65 (1.02, 2.67)	0.044
Residual Syntax Score	1.03 (1.0, 1.05)	0.014	2.04 (1.09, 3.8)	0.025
Diabetes mellitus	1.82 (1.14, 2.9)	0.012	1.59 (1.0, 2.57)	0.056
Arterial Hypertension	1.69 (0.98, 2.91)	0.059	1.37 (0.78, 2.4)	0.273
Age (years)	1.03 (1.0, 1.05)	0.029	1.02 (0.99, 1.04)	0.231
Female sex	1.53 (0.9, 2.6)	0.12	1.26 (0.7, 2.26)	0.441

*CI* indicates confidence interval, *OSA* Obstructive Sleep Apnea

## Discussion

The findings of the present study can be summarized as follows: 1) OSA with ACS patients were associated with a 2-fold higher risk of MACCEs than in those non-OSA; 2) compared to the non-OSA patients, OSA with ACS patients presented more severe coronary artery disease; 3) the Residual SYNTAX Score was able to stratify the OSA patients with ACS; 4) incomplete revascularization was associated with high clinical events in the OSA patients with ACS.

OSA has been advocated as a new independent risk factor for coronary artery disease [4–6]. However, the link between cardiovascular events and OSA is still controversial [10] because of the following reasons: (1) most of the studies were observational ones for both ethical and logistical reasons. Symptomatic cases will benefit from long-term CPAP, which was challenging to make OSA patients free from treatment; (2) CPAP treatment adherence of patients was important to guarantee the beneficial effect [17]. Studies showed beneficial effects of CPAP only in those who used CPAP for 4 to 6 h/night or longer [9, 18]; 3) OSA treatment effect might be confounded by a treatment bias, those with severe symptoms might also adhere to diet, exercise and other drugs better than those without or mild symptoms. Thus, the net beneficial effect of OSA treatment was difficult to draw [4]. Therefore, compelling causal evidences between OSA and cardiovascular events are still lacking. In the prospective study including a large Chinese ACS population in real-world, the prevalence of OSA was approximately 50%, which is similar to that reported in previous studies [19]. We demonstrated that OSA was associated with a 2-fold increased risk of MACCEs compared with the non-OSA patients, which was mainly driven by unstable angina pectoris.

Elucidating the effects of OSA on the coronary angiographic characteristics is the fundamental pathologic substrate to understand the underlying effects on cardiovascular events. OSA was reported to be associated with more multi-vessel disease, more severe coronary artery calcification, as well as more ST segment depression [20–22]. Our findings were consistent with the literature: higher frequency of three-vessel disease, two-vessel disease were observed in the OSA patients. We added further evidence that the OSA patients with ACS presented with more chronic total occlusion lesions, which finally led to more stent implantation comparing with the non-OSA patients. The possible mechanisms might include vascular endothelial injury, insulin resistance, sympathetic activation, neurohumoral changes, inflammation, oxidative stress, dyslipidemia through chronic intermittent hypoxia [23–25]. The disorders of metabolic function mentioned above ultimately induced or accelerated the process of coronary artery disease.

Several scoring systems have been developed to evaluate the complexity of coronary artery disease. Gensini score was deprived for each coronary stenosis on the basis of the degree of luminal narrowing and its topogtaphic importance [26]. SYNTAX score takes into account not only the luminal narrowing, but also other anatomic aspects, including bifurcation, trifurcation lesions, calcification, tortuosity, thrombus, and occlusions etc. Residual SYNTAX Score deriving from SYNTAX Score was used to quantify the residual burden of coronary artery disease after revascularization [12–14]. ICR was a surrogate marker of patients with more anatomically complex baseline disease. We found in the present study that ICR was more frequently observed in the OSA patients with ACS compared with those non- OSA. This implicates complex coronary lesion in the OSA patients and would predict worse prognostic events.

Previous studies have demonstrated that incomplete revascularization after either PCI or CABG was at a high risk of MACCEs in heterogeneous patients including ACS, STEMI, NSTEMI, multi-vessel disease and complex lesion [27–31]. However, studies evaluating the impact of rSS in patients with OSA and ACS have not yet been elucidated. We assessed for the first time the prognostic effect of rSS on clinical outcomes in a large prospective study. We found that ICR was associated with a 2-fold higher risk of MACCEs compared with CR in the OSA group. The OSA patients had a similar 1-year MACCEs as all the non-OSA if the revascularization was complete. Similarly, there was also no significant difference in the 1-year MACCEs rate in the OSA and the non-OSA patients if the revascularization was complete in both groups.

## Limitations

There were several limitations in the study. Firstly, the non-randomized design of the study will preclude the conclusion. Secondly, the follow-up was relatively short to observe the incidence of long-term events. Thirdly, the sample size was relative small in subgroup analysis. Fourthly, rSS was based on angiographic interpretation that has inherent limitations [32]. However, rSS was assessed by consensus of well-trained analysts with good reproducibility. In addition, the strategy of PCI or CABG in the present study was decided by a heart team consisting of cardiac surgeons and cardiologists in one center, this reduces the potential confounder of operator discretion. Fifthly, residual SS was not calculated based on functional evaluation with fractional flow reserve, however, the latter has not been implemented routinely in multi-vessel disease in China.

## Conclusions

ACS patients with OSA and ICR have a high rate of MACCEs at 1 year. In contrast, the prognosis of ACS patients with OSA but CR is favorable and similar to patients without non-OSA. Adequate level of revascularization is recommended to optimize clinical outcomes in ACS patients with OSA.

## Abbreviations

ACS: Acute coronary syndrome
AHI: Apnea hypopnea index
CABG: Coronary artery bypass graft
CR: Complete revascularization
CTO: Chronic total occlusion
ICR: Incomplete revascularization
MACCEs: Major adverse cardiovascular and cerebrovascular events
OSA: Obstructive sleep apnea
PCI: Percutaneous coronary intervention
rSS: residual SYNTAX score
SS: SYNTAX score
